# The role of shifted television viewing on earlier bedtimes in the streaming era

**DOI:** 10.1016/j.socscimed.2025.117977

**Published:** 2025-03-18

**Authors:** Jess M. Meyer, Jarron M. Saint Onge, Catherine F. Siengsukon

**Affiliations:** a Department of Population Health, School of Medicine, University of Kansas Medical Center, Mail Stop 1008, 3901 Rainbow Blvd., Kansas City, KS, 66160, United States; b Department of Sociology, College of Liberal Arts & Sciences, University of Kansas, Fraser Hall, Room 716, 1415 Jayhawk Blvd., Lawrence, KS, 66045, United States; c Department of Physical Therapy, Rehabilitation Science, and Athletic Training, School of Health Professions, University of Kansas Medical Center, Mail Stop 2002, 3901 Rainbow Blvd., Kansas City, KS, 66160, United States

**Keywords:** Sleep, Television, Bedtime, Health behavior

## Abstract

**Objectives:**

To investigate the extent to which changes in the timing of television viewership explained shifts in bedtimes between 2003 and 2019, a period in which several major video streaming services began operation.

**Methods:**

We used time diaries from U.S. adults in the American Time Use Survey (N = 196,569) to examine changes in the timing of bedtimes and television viewership, disaggregated by age. We used mediation analysis to identify the extent to which changes in evening viewership timing explained recent shifts in bedtime.

**Results:**

From 2003 to 2019, both bedtimes and cessation of evening television viewing shifted earlier among adults under age 65. When adjusting for work hours, sociodemographic characteristics, and interview timing, these adults stopped watching evening television 10–30 min earlier in 2019 relative to 2003. The largest television shift was observed among 18–29-year-olds on weekdays, who stopped watching at 10:12 p.m. in 2003 and 9:42 p.m. in 2019. The shift in viewership timing accounted for nearly 45 % of the shift to earlier bedtimes on weekdays and 49 % on weekends/holidays over this period.

**Conclusions:**

Among adults under age 65, findings show that earlier television viewing cessation statistically accounted for earlier bedtimes observed in the streaming era. Although we are unable to directly link changes in streaming to bedtimes, observed shifts in television viewing suggest streaming may allow people to schedule viewing at times that avoid conflict with sleep. Further, attempts to understand television viewing habits and shifting time use offer important social insights into sleep health.

## 1. Introduction

The introduction of television into the household in the mid-20th century led to significant changes in how people allocated time (Robinson and Godbey, 1999), with important implications for health. Spending more time watching television has been linked to higher risk of cardiovascular disease and type 2 diabetes (Grøntved and Hu, 2011). Recent expansion of accessible and reliable digital video streaming services represents another potential major shift in time allocation and health. In the past, a person may have been limited in video media consumption opportunities due to fewer available devices in the household, show release schedules, or prohibitive costs of cable, video rentals, or data usage. Present day, the ability to consume video media is now nearly limitless for many, potentially affecting the quantity of media consumed. In addition, these changes in how people consume video media outside of movie theaters (hereafter referred to as “television viewing”) have potential consequences for the timing of *when* they watch, with the timing of television viewing implicated in sleep patterns (Basner and Dinges, 2009; Basner et al., 2007, 2014; Hamermesh et al., 2008). Yet, there remains relatively limited evidence examining how population-level changes in viewing patterns over time are associated with changes in adults’ sleep schedules.

Empirical evidence on how television viewing is related to sleep is important because streaming technologies could either be health compromising or health promoting for sleep timing. Watching television is the most common activity in the 2 h before bed, underlining the connection between television viewing and bedtime (Basner and Dinges, 2009). Accordingly, increased television viewing has the potential to negatively impact sleep by pushing bedtimes later, as 24-h availability of high-quality programming might increase viewing late at night (Wider, 2018). The availability of multiple seasons of a television series may also lead to higher levels of binge-watching or watching several episodes of a series at the expense of an earlier or more regular bedtime (Vaterlaus et al., 2019). In addition, binge watching is associated with greater insomnia symptoms and poorer sleep quality via heighted cognitive arousal at bedtime (Exelmans & Van den Bulck, 2017). Furthermore, watching videos on computers and mobile devices may lead people to actively engage more with social media or work-related tasks on those devices, further contributing to sleep delays (Meyerson et al., 2023).

Conversely, increased control over television viewership timing might promote healthier sleep schedules. Availability of content over the entire 24-h day may facilitate viewing at times that do not interfere with sleep. Evidence from college students suggests that the frequency of “appointment viewing,” (i.e., the more traditional pattern of watching programming at the time it is broadcast), has declined in recent years (Rubenking and Bracken, 2021). Although this has co-occurred with an increase in binge watching, “serial viewing,” or watching a series at one’s own pace, has also increased (Rubenking and Bracken, 2021). A 2018 analysis of American Time Use Survey respondents who were in school full-time, employed, or retired found that bedtimes moved earlier from 2003 to 2016, with a decreasing proportion of respondents watching television just before bed (Basner and Dinges, 2018). Earlier bedtimes, in turn, may explain recent increases in sleep duration (Basner and Dinges, 2018). Thus, while television viewing, particularly in the evening, may negatively impact sleep (Basner and Dinges, 2009), recent technological changes in television viewing options may positively impact sleep schedules by facilitating people’s ability to set their own viewing schedules and time shift (Wajcman, 2008) viewing. However, the exact link between recent changes in television viewing and sleep timing remains unclear.

Changes in television and sleep behaviors may potentially vary by age due to either differences in technology uptake or differences in time allocation. Regarding technology uptake, in 2017, over half of U.S. adults ages 18–29 years reported online streaming as their primary method of viewing television (Pew Research Center, 2017). However, this proportion was 37 % among adults ages 30 to 49 and 10 % among adults ages 50 to 64 (Pew Research Center, 2017). Among adults 65 and older, only 5 % reported streaming as their primary method of viewing television (Pew Research Center, 2017). Given this reduced streaming uptake at older ages, we might expect the impacts of streaming introduction to have been more muted among older adults. Regarding time allocation, on average, adults 65–75 years old watch more television per day than those 55–64 years old, and, among older adults, those who are not in the labor force allocate more time to television viewing (Flood and Moen, 2015; Krantz-Kent and Stewart, 2007). However, although retirees may have more flexibility in their schedules to accommodate viewing preferences by shifting time use, qualitative work has emphasized the role of relative consistencies in structured behavioral routines on simplifying and motivating healthy behaviors among retirees (Ekerdt and Koss, 2016).

In the present study, we investigated changes in the timing of television viewership and bedtimes among adults living in the United States from 2003 to 2019, a period in which the U.S. experienced an expansion in the availability and use of video streaming services. YouTube, for example, was founded in 2005 (Anderson, 2015), and Netflix introduced video streaming in 2007 (Helft, 2007; Kyncl, 2017). In 2009, only 10 % of U.S. households had a subscription to a paid video streaming platform, but by 2017, a majority of U.S. households (55 %) did (Center for Technology, 2018). Further, given differences in streaming use by age (Pew Research Center, 2017), we tested whether there were differences by age in how the timing of television viewership and bedtimes changed over this period.

First, we examined how bedtimes changed from 2003 to 2019, by age group. Second, given that television viewing in the *evening* (versus earlier in the day) is most likely related to bedtime (Basner and Dinges, 2009), we analyzed the magnitude of changes in evening television viewing end times—by age. Next, we tested the extent to which changes in the time people stopped watching evening television explained observed changes in bedtimes, among age groups that demonstrated changes. Finally, we examined how the distribution of television viewing shifted between weekdays and weekends/holidays and across the 24-h day, among age groups that demonstrated shifts in bedtimes over this time period.

## 2. Methods

We used data from the American Time Use Survey (ATUS), a nationally representative sample drawn from a subsample of households that participated in the Current Population Survey (CPS) and is representative of the civilian, noninstitutionalized U.S. population ages 15 and older (Bureau of Labor Statistics, 2023). The ATUS is cross-sectional and collects time diaries 2–5 months after CPS completion via telephone interview about respondents’ activities in one 24-h period ranging from 4:00 a.m. the day prior to 4:00 a.m. on the interview day (Bureau of Labor Statistics, 2023). Adults selected to participate in the ATUS were sent mailed information that provided advance notice of their interview date (and the possibility to reschedule that date) and informed them that they would be asked about their time use and “how you spent the past 24 h” (Bureau of Labor Statistics, 2023).

For the ATUS time diary, interviewers used computer-assisted telephone interviewing to record the duration/timing of each activity as reported by the respondent, with the capability to record responses at the level of the minute (or hour) (Bureau of Labor Statistics, 2012, 2023, 2024). ATUS data collection focused on primary (versus simultaneous, secondary) activities (with exceptions for childcare, eldercare [since 2011], and, in an Eating and Health Module for specific years, eating and drinking, which could be recorded as simultaneous, secondary activities) (Bureau of Labor Statistics, 2012, 2023, 2024). In ATUS data production, two coders independently assigned activity codes to time diary activities, following ATUS guidelines; coding discrepancies were addressed by an adjudicator (Bureau of Labor Statistics, 2023).

We analyzed time diaries starting in 2003 (the first ATUS year) and ending in 2019 due to potential time use and data changes related to the COVID-19 pandemic (Flood et al., 2022). The dataset was generated by the ATUS Extract Builder (Flood et al., 2023). Beginning with all respondents from 2003 to 2019 (N = 210,586), respondents were excluded from our sample if they were: <18 years old (N = 8,741); lacking a valid bedtime (N = 4,982); or missing covariate data (N = 294). This resulted in an analytical sample of 196,569 respondents. The sample size, disaggregated by year, is available in Supplementary Table A1 of online Appendix A. At the start of the observation period, in 2003, the full sample was 47.7 % male, 72.6 % Non-Hispanic White, 12.3 % Hispanic, 10.7 % Non-Hispanic Black, and 4.5 % “Other,” with a mean age of 45.5 (standard deviation 23.5 years) (Table A2). By the end of the observation period, in 2019, sample demographics varied slightly. The full sample was 48.1 % male, 65.6 % Non-Hispanic White, 16.4 % Hispanic, 11.7 % Non-Hispanic Black, and 6.3 % “Other,” with a mean age of 47.9 (standard deviation of 15.6 years). In analyses of evening television viewing end times and bedtimes, we further restricted the sample to respondents who stopped watching television at 5:00 p.m. or later on the diary day (N = 148,378; descriptive statistics for 2003 and 2019 in Table A3).

### 2.1. Television viewing measures

Respondents were considered to be viewing television when their primary activity was ATUS activity code 120303 (“Television and movies (not religious)”) or 120304 (“Television (religious)”) (Flood et al., 2023). ATUS coding rules specify that watching video in a home falls under the code 120303; watching movies in a movie theater is not included in this definition (Flood et al., 2023; Krantz-Kent, 2018). *Evening television viewing end time* was defined as the time at which the last episode of evening television viewing ended. *Amount of television viewed* represented the number of hours spent watching television over the full diary day. *Ratio of weekend/holiday to weekday television hours viewed* represented the amount of television viewed on weekends/holidays divided by amount viewed on weekdays. Because respondents only completed either a weekend/holiday or weekday time diary, this was calculated at the aggregate level across all respondents for each year. We also constructed a dichotomous indicator of *hour-specific viewing* for each hour of the day that denoted whether respondents viewed any television during each respective hour.

### 2.2. Bedtime measure

*Bedtime* was defined as the start of the longest sleep episode (Burgard and Ailshire, 2013) that began after 5:00 p.m. Respondents who did not have a sleep episode that started after 5:00 p.m. were excluded due to invalid bedtime. (Approximately 2.5 % of respondents meeting age-related inclusion criteria were excluded for this reason.)

### 2.3. Additional predictor variables

*Year* was the year of the interview and was centered at 2003 in regression models to facilitate interpretation of coefficients in supplementary tables. Given our focus on bedtimes, *weekdays* were defined as (non-holiday) Sunday through Thursday, and *weekends/holidays* were defined as Friday, Saturday, and holidays (e.g., major holidays such as Fourth of July.) For weekday-specific regression models, covariates included *day of the week* (Sunday, Monday, Tuesday, Wednesday, or Thursday), and weekend/holiday model covariates included *type of day* (whether the diary was completed for a non-holiday Friday, non-holiday Saturday, or holiday).

Additional covariates included the following: *region* (South, Northeast, Midwest, or West); *age* (centered at 18, both linear and squared); *sex* (female or male); *race/ethnicity* (coded as Non-Hispanic White, Hispanic, Non-Hispanic Black, or Other; derived from available CPS proxy or self-report or imputed data in ATUS—see (Bureau of Labor Statistics, 2023; Flood et al., 2023; U.S. Census Bureau, 2019) for details) as a potential social determinant of bedtimes (Combs et al., 2021); *education* (less than high school, high school, some college or associate’s, or college or advanced degree); *number of children in the household* (zero, one, two, or three or more); *partnership status* (living with spouse, living with unmarried partner, or not living with partner); and *nativity* status (whether born in the U.S.) (Seicean et al., 2011). Covariates also included a measure of *season* (spring, summer, fall, or winter) and *work hours,* the number of hours spent in work-related activities that day, which does not include educational or volunteer activities.

### 2.4. Analysis

First, to investigate changes in bedtimes from 2003 to 2019, we examined descriptive statistics of the average bedtime by year, disaggregated by age group: 18–29 years old, 30–49 years old, 50–64 years old, and 65 years old and above. We separately analyzed weekdays and weekends/holidays to adjust for potential differences in schedule constraints and television viewership timing (Krantz-Kent, 2018), which may affect the flexibility of television viewership and bedtimes to shift over time. We used Stata’s “*lincom*” command to assess the statistical significance of differences in bedtimes between 2003, the start of our observation period and 2019, the end of our observation period.

Second, we used multivariable regression to predict change in both bedtimes and television viewing end times, restricting our sample to individuals who watched evening television on the diary day. In a pooled sample (not disaggregated by age), we compared unweighted models including linear, quadratic, and cubed terms of *years since 2003*, using Bayesian Information Criterion (BIC), Akaike’s Information Criterion (AIC), and likelihood ratio test statistics to guide model selection. In final models disaggregated by age, we included covariates for region, season (Krantz-Kent, 2018), day/type of day, age, age squared, sex, race/ethnicity, education, number of household children, partnership status, nativity (Seicean et al., 2011), and hours worked (in both weighted and unweighted models).

Third, we analyzed the association of television viewing end time with bedtimes for the subset of respondents whose age group displayed statistically significant shifts in bedtimes and television viewing end times. Pooling these respondents, we ran two regression models. Model 1 controlled for the same set of covariates mentioned above, in addition to hours spent viewing television. Model 2 added the time at which respondents stopped watching television. Next, we used the “*sgmediation2*” package (Mize, 2022) to formally test the extent to which television viewing end time mediated any statistically significant changes in bedtimes.

Finally, to further investigate shifts in television viewership timing from 2003 to 2019, we examined changes in the distribution of television viewership between weekdays and weekends/holidays and across the 24-h day. We focused these analyses on age groups that had displayed statistically significant shifts in bedtimes, and we included individuals who had not watched evening television on the diary day. We first examined the ratio of mean hours of television watched on weekends/holidays to mean hours watched on weekdays. We regressed this ratio on year to assess linear change over time. To investigate how changes in weekday versus weekend/holiday viewing were associated with this ratio, we also examined how the average amount of television watched per day changed over time on weekdays versus weekends/holidays. Wald tests were used to assess statistical significance of differences in average hours of television viewed between the beginning and end of the observation period. Second, we compared 2003 to 2019 in the proportion of respondents with *hour-specific viewing* across the 24-h day. Wald tests were used to assess statistical significance of differences in proportions.

With one exception, main analyses used probability weights to calculate point estimates and, where necessary, successive difference replicate weights to calculate standard errors, with Stata/SE 18.0 or 18.5. The model that regressed weekend/holiday-to-weekday television ratio used year as the unit of analysis and therefore did not incorporate individual sampling weights (though did use weights to generate ratio estimates). For calculating marginal effects and predicted values based on regression models, the “*mgen*” (Long and Freese, 2014) command was used, with covariates held at means.

## 3. Results

### 3.1. Change in bedtimes by age

**Fig. 1.**
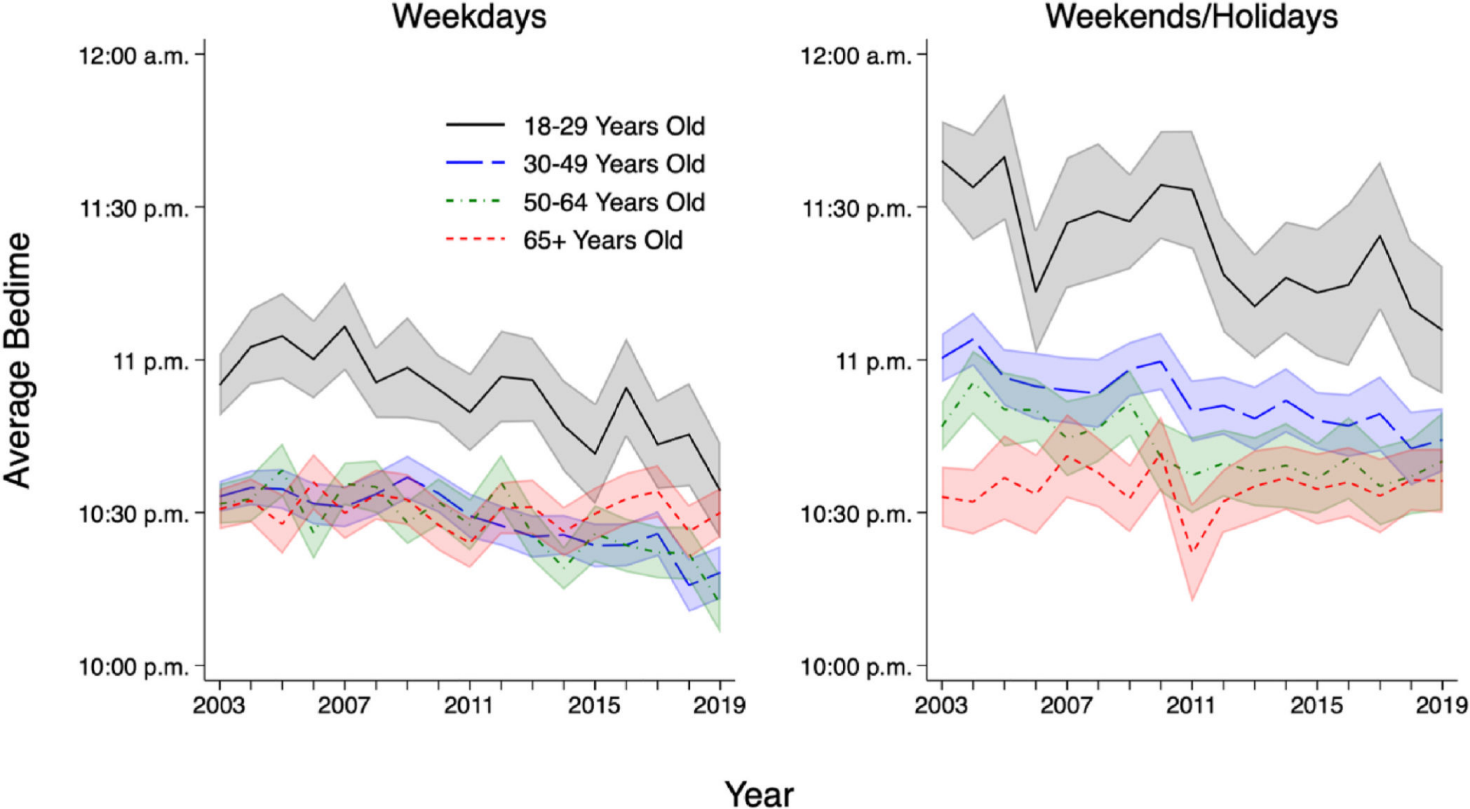
Average bedtime by year and age, years 2003–2019 (adult ATUS respondents with valid bedtime). Note: Generated using sampling weights. Total N = 196,569 (126,587 for weekdays and 69,982 for weekends/holidays). Shaded areas show 95 % confidence intervals. Source: American Time Use Survey, 2003–2019.

Descriptive statistics in Table A2 confirmed that when pooling across all ages, bedtimes were roughly 15 min earlier in 2019, the end of our observation period, compared to 2003, the beginning of observation period. Fig. 1 shows the average bedtime across the full observation period, broken down by age group. For the youngest adults in the sample, ages 18–29 years old, bedtimes clearly shifted earlier from 2003 to 2019. On weekdays, 18–29-year-olds’ average bedtime was 10:55 p. m. in 2003 but by 2019 had shifted over 20 min earlier, to 10:34 p.m. On weekends/holidays, 18–29-year-olds’ average bedtime shifted over a half-hour earlier, from 11:39 p.m. in 2003 to 11:06 p.m. in 2019.

For adults 30–49 and 50–64 years old, bedtimes appeared to shift earlier as well, though in general, shifts in these age groups appeared subtler than those observed among the youngest adults. Among adults 30–49 years old, compared to 2003, by 2019 bedtimes were 15 min earlier on weekdays and 16 min earlier on weekends/holidays. For adults 50–64 years old, compared to 2003, by 2019 bedtimes were 20 min earlier on weekdays and 7 min earlier on weekends/holidays (the difference between 2003 and 2019 for weekends/holidays was not statistically significant, with *p* = 0.230). In contrast to findings for younger adults, for the oldest adults in the sample, ages 65 and older, bedtimes did not shift earlier over the observation period, on either weekdays or weekends/holidays.

### 3.2. Do changes in television viewing end time explain changes in bedtime?

#### 3.2.1. Changes in bedtimes and television viewing end times by age

Paralleling findings from the full sample, descriptive statistics in Table A3 show that when pooling across all ages, for the subset of respondents who watched television in the evening, bedtimes were roughly 15 min earlier in 2019 than in 2003. In this subset of respondents, the time at which people stopped watching evening television was also earlier in 2019 relative to 2003. On average, people stopped watching television in the evening at 10:10 p.m. in 2003—versus 9:57 p.m. in 2019.

Fit statistics from unweighted regression models predicting bedtimes and television viewing end times (pooled across all ages) helped discern how best to model change over time in these outcomes (results in Tables A4 and A5 for bedtimes and television viewing end times, respectively). Fit statistics suggested a linear decrease in bedtimes and television viewing end times on weekends/holidays—or in other words, that bedtimes and television viewing end times shifted earlier in a linear fashion over time. In contrast, for weekdays, results overall suggested that the best approach was to model a curvilinear trend of increasingly negative change on weekdays—in other words, bedtimes and television viewing end times shifted increasingly earlier over the observation period.

**Fig. 2.**
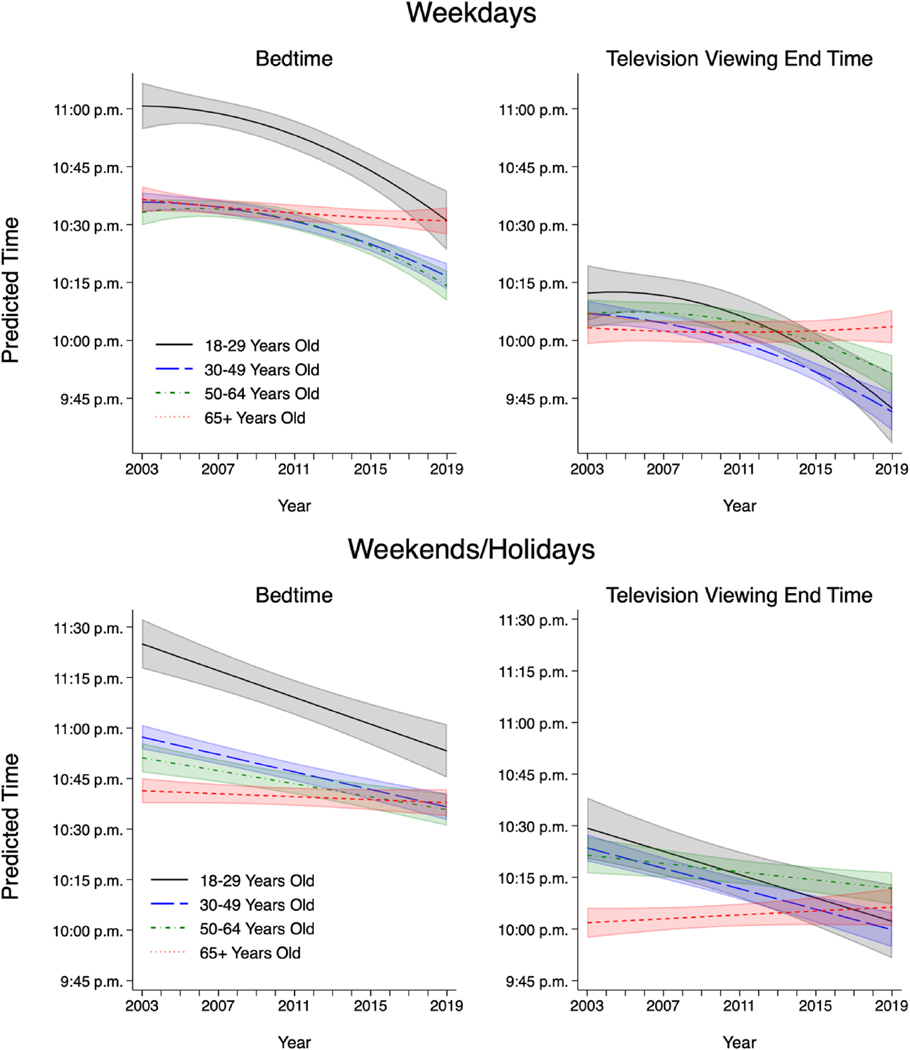
Predicted bedtime and television viewing end time by age and year among adult ATUS respondents with valid bedtime who viewed evening television, years 2003–2019. Note: Generated using sampling weights in models disaggregated by age. N = 148,378 total (N’s broken down by age group and type of day in Table A6). Shaded areas show 95 % confidence intervals. Covariates held at their means within each age group. Models adjust for age (and age squared), region, season, sex, race/ethnicity, education, number of children in the household, partnership status, nativity, hours worked on the diary day. The weekday model controls for day of the week (Sunday, Monday, Tuesday, Wednesday, or Thursday), and the weekend/holiday model controls for whether the diary day was a non-holiday Friday, non-holiday Saturday, or holiday. Source: American Time Use Survey, 2003–2019.

Fig. 2 displays predicted bedtimes and television viewing end times based on multivariable regression models among respondents who watched television in the evening, broken down by age. Within each age group, covariates were held at their means. Selected coefficients for these models are shown in Table A6. On weekdays, among adults 18–29, 30–49, and 50–64 years old, both bedtimes and television viewing end times shifted increasingly earlier from 2003 to 2019, by between 15 and 30 min. The largest weekday shift in the predicted end time of television viewing was observed among adults 18–29 years old, who shifted from 10:12 p.m. in 2003 to 9:42 p.m. in 2019. In contrast, neither bedtimes nor television viewing end times experienced a significant trend of shift among adults 65 years old and older. On weekends/holidays, a similar pattern was observed: bedtimes and television end times shifted earlier over time among adults ages 18–29, 30–49, and 50–64 years old, by between roughly 10–32 min. Again, for adults 65 years of age and older, neither bedtimes nor television viewing end times displayed a statistically significant trend of change over this time period.

We conducted an ancillary test of the statistical significance of differences by age group in change over time, using a pooled model containing all ages and interacting change over time with each age category (using 18–29-year-olds as the reference). Results demonstrated that on both weekdays and weekends/holidays, patterns of change in bedtimes and television viewing end times significantly differed between respondents 18–29 years old compared to respondents 65 and older, confirming our results from disaggregated models. (On weekends/holidays only, patterns of change also significantly differed between the 18–29-year-old and 50–64-year-old age groups).

#### 3.2.2. Did changes in television viewing end time mediate changes in bedtime?

**Fig. 3.**
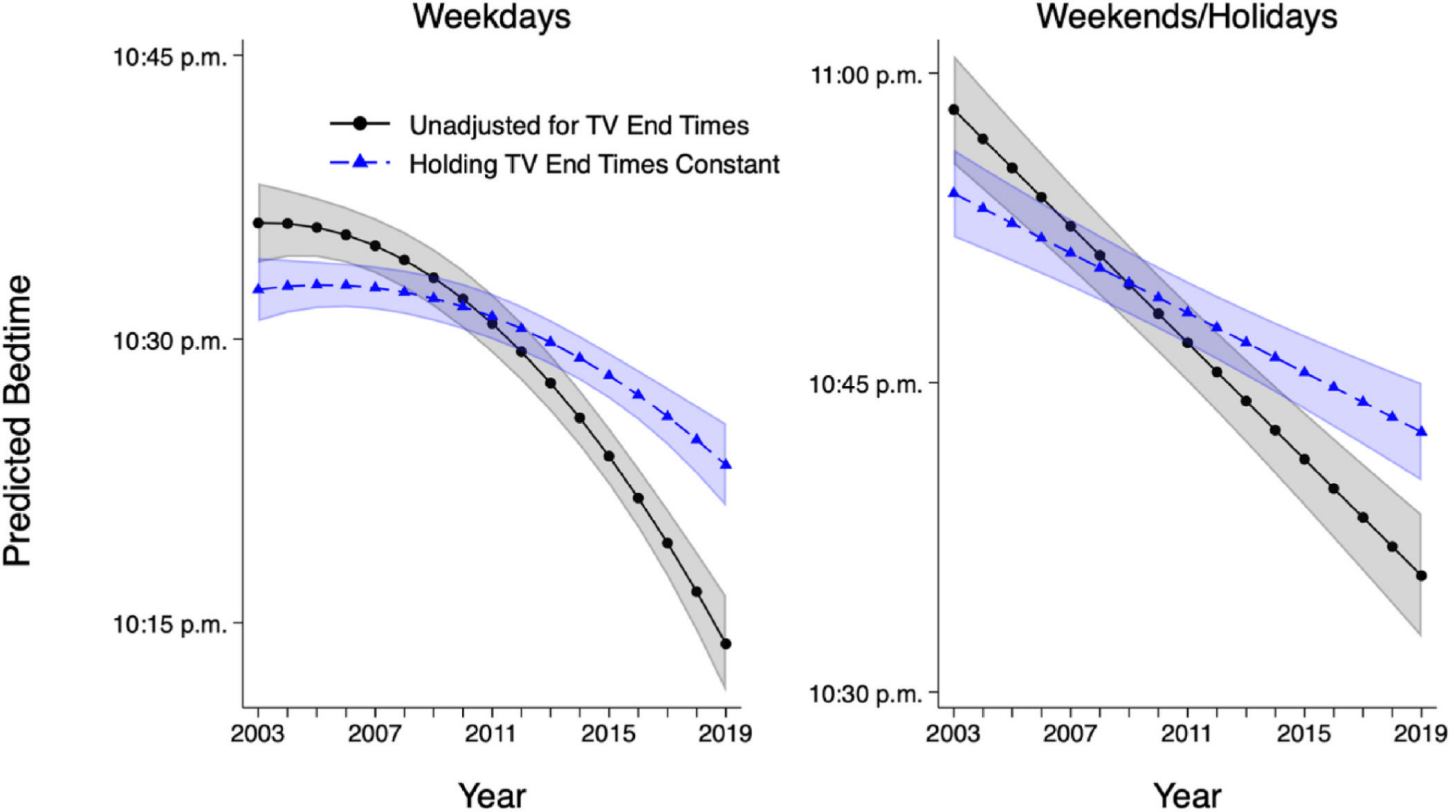
Predicted bedtime by year, years 2003–2019 (ATUS respondents 18–64 years old with valid bedtime who viewed evening television). Note: Generated using sampling weights. N = 73,750 for weekdays and 39,901 for weekends and holidays. Shaded areas show 95 % confidence intervals. Covariates held at their means. Model 1 adjusted for region, season, age (and age squared), sex, race/ethnicity, education, number of children in the household, partnership status, nativity, hours worked on the diary day, and amount of television viewed on the diary day. Weekday model controlled for day of the week (Sunday, Monday, Tuesday, Wednesday, or Thursday), and weekend/holiday model controlled for whether the diary day was a non-holiday Friday, non-holiday Saturday, or holiday. Model 2 added a covariate for television viewing (TV) end time. Source: American Time Use Survey, 2003–2019.

Fig. 3 shows predicted bedtimes based on multivariable regression among respondents who watched evening television (selected coefficients shown in Table A7). The sample is restricted to respondents under 65 years old (i.e., age groups that demonstrated changes over the observation period) and is presented as pooled across ages for ease of visualization. Results from baseline models are shown in lines with circles; results from models that adjust for television viewing end times are shown in lines with triangles. On weekdays, the rate of linear change in bedtimes (represented by the coefficient “Years Since 2003” in Table A7) was not statistically significant. However, the rate of change in bedtimes with each passing year grew increasingly negative (with bedtimes shifting increasingly earlier) over time. When not adjusting for television viewing end time, by the end of the observation period in 2019, the rate of change in bedtimes reached a shift of 2.8 min earlier per year. Holding covariates at their means, the predicted bedtime on weekdays moved from 10:36 p.m. in 2003 to 10:14 p.m. in 2019. In the model unadjusted for television viewing end time, weekend/holiday bedtimes shifted earlier in a linear fashion, at a rate of 1.4 min per year. Holding covariates at their means, relative to 2003, the predicted bedtime had moved over 22 min earlier to 10:36 p.m. in 2019.

Adjusting for the time at which respondents stopped watching evening television substantially flattened the patterns of change in bedtimes (Fig. 3). In other words, when we held television viewing end times constant, we did not observe the same degree of shift in bedtimes. Additional, formal mediation analyses pooled across all age groups (Table A8) showed that on weekdays, nearly 45 % of the curvilinear (quadratic) change in bedtimes was explained by television viewing end time (statistically significant Sobel test at *p* = 0.001). For weekends/holidays, nearly 49 % of the linear change in bedtimes was accounted for by television viewing end time (Sobel test *p* = 0.000 [rounded to three digits]).

### 3.3. Changes in the distribution of television viewing

#### 3.3.1. Did the distribution of television viewing shift between weekdays versus weekends/holidays?

**Fig. 4.**
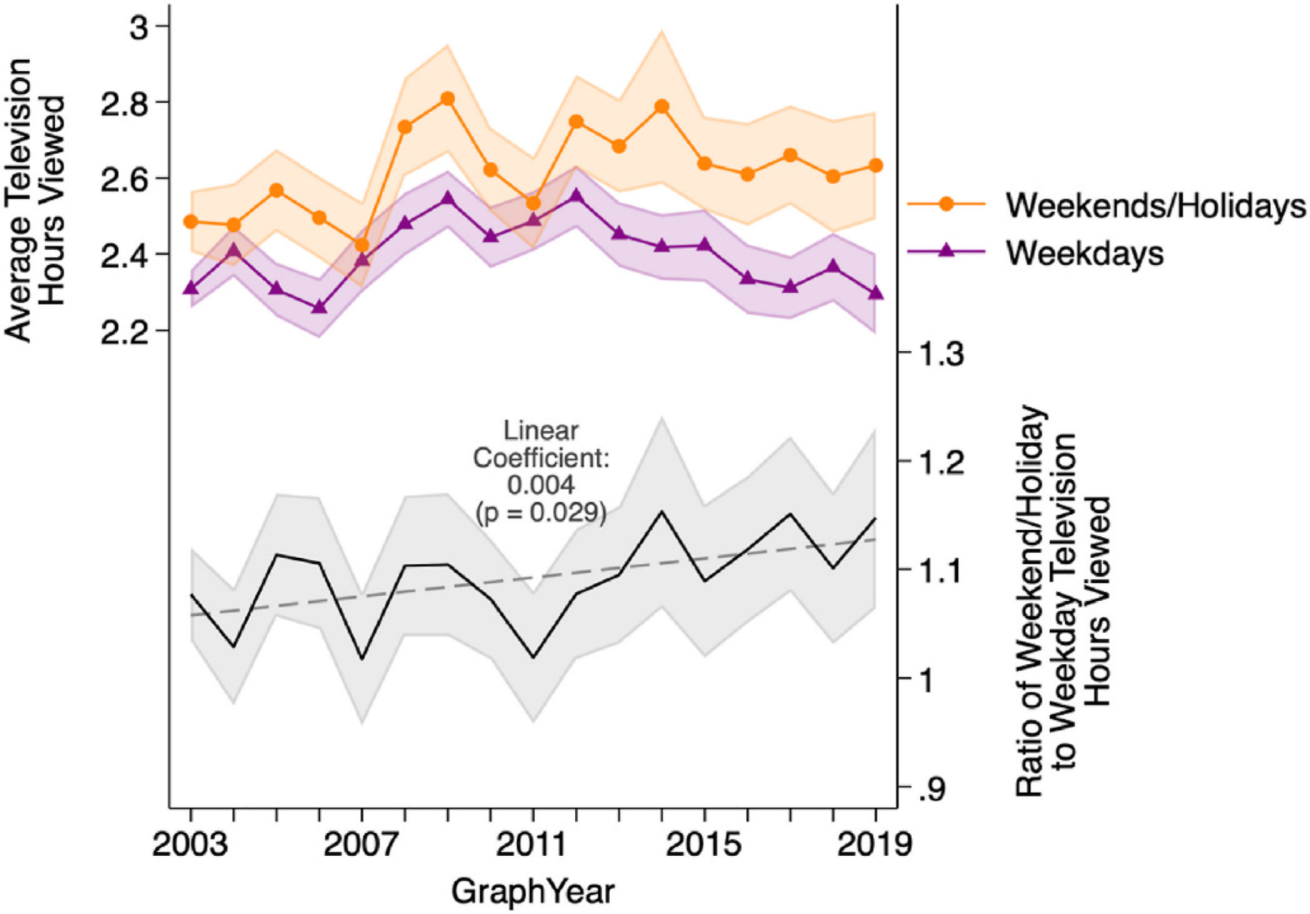
Television viewed by year, years 2003–2019 (ATUS respondents 18–64 years old with valid bedtime). Note: Solid lines show descriptive statistics generated using sampling weights at the individual level. Shaded areas show 95 % confidence intervals. N = 155,330 (99,623 on weekdays and 55,707 on weekends/holidays). Dashed line shows linear trend generated from regression with year as the unit of analysis. Source: American Time Use Survey, 2003–2019.

The bottom portion of Fig. 4 shows the ratio of average amount of television watched on weekends/holidays relative to the average amount of television watched on weekdays, by year, for adults under age 65. Because respondents completed only one time diary (i.e., either on a weekday or weekend/holiday), this ratio was calculated at the aggregate level. For adults younger than age 65, the ratio of the mean amount of time spent viewing television on weekends/holidays to the mean amount of time spent viewing television on weekdays rose from 1.08 in 2003 to 1.15 in 2019. In a model regressing this ratio on year, the linear increase was statistically significant (*p* = 0.029). Thus, as time passed, the distribution of television viewing shifted more towards weekends/holidays, on average. However, we note that when disaggregating results by age (Fig. A1), a clear trend of change was more difficult to discern.

The top portion of Fig. 4 examines the dynamics of this shift in greater detail by displaying how average television hours changed over time, separately for weekdays and weekends/holidays, with averages calculated across individuals. In the first portion of our observation period, there appeared to be a general trend of increase in the amount of time people spent viewing television, on both weekdays and weekends/holidays. However, this increase stopped around roughly the middle of our observation period, at which point the amount of television viewed on weekends/holidays appeared to somewhat plateau, and the amount of television viewed on weekdays more clearly declined. Whereas the amount of television viewed on weekends/holidays remained elevated in 2019 compared to 2003 (*p*-value of difference = 0.068), by 2019, the amount of television viewed on *weekdays* was similar to 2003 levels.

#### 3.3.2. Did the distribution of television viewing shift across the day?

**Fig. 5.**
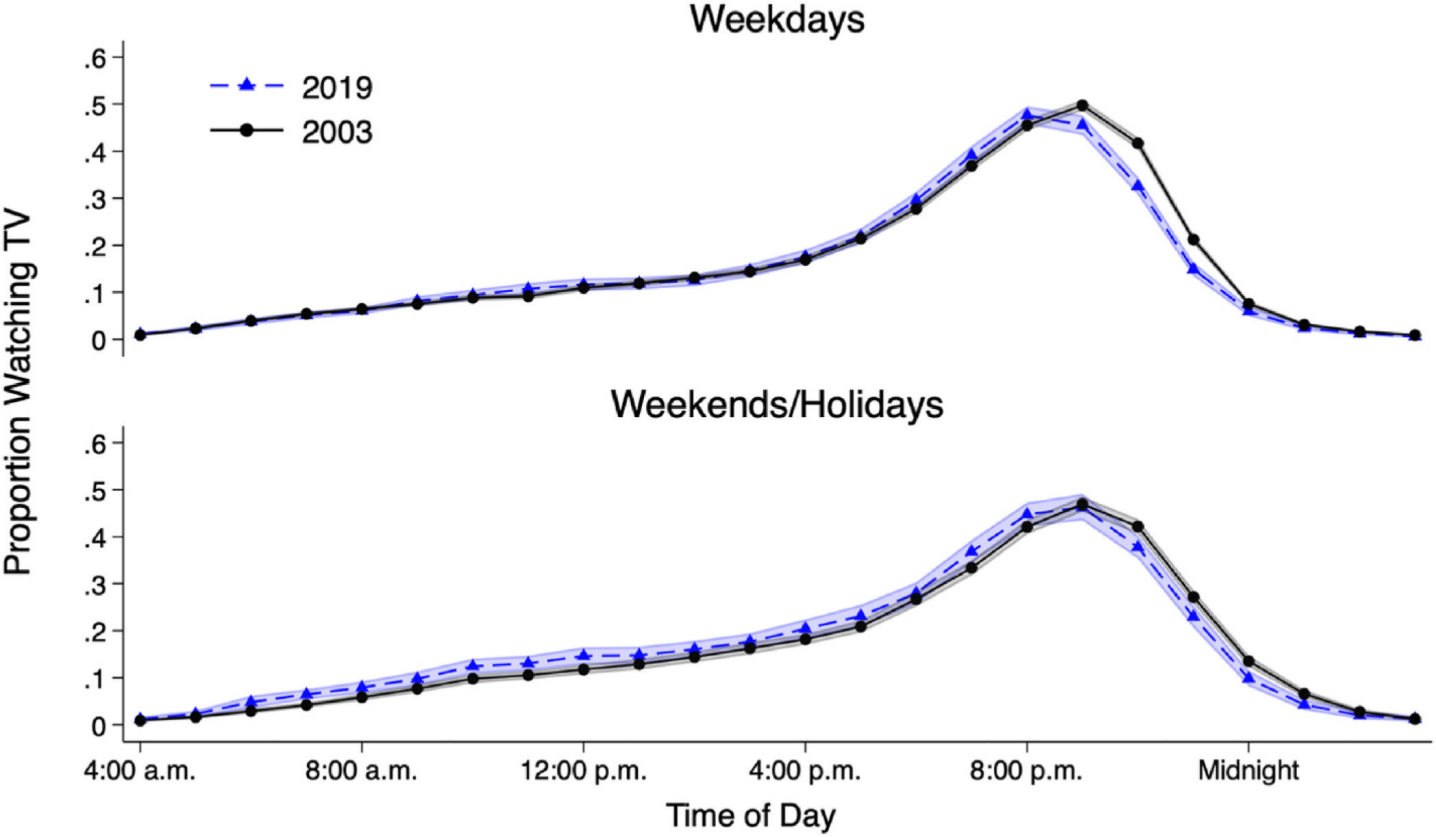
Proportion that viewed television within each hour of the day, year 2003 versus 2019 (ATUS respondents 18–64 years old with valid bedtime). Note: TV = television. Shaded areas show 95 % confidence intervals. Generated using sampling weights. N = 22,212 individuals (N = 10,115 for weekdays in 2003, 5,669 for weekends/holidays in 2003, 4,158 for weekdays in 2019, and 2,270 for weekends/holidays in 2019). Source: American Time Use Survey, 2003; 2019.

Fig. 5 compares the proportion of respondents watching television at each hour of the day in 2003–2019. On weekdays, a lower proportion of respondents viewed television from 9:00 p.m. to just before 1:00 a.m. in 2019 than in 2003 (*p* < 0.01 in Wald test). This difference reaches a peak in the 10:00 p.m. hour, when approximately 42 % of respondents watched television in 2003, versus 33 % in 2019. In contrast, from the beginning of the 7:00 p.m. hour to the end of the 8:00 p.m. hour, a higher proportion of respondents watched television in 2019 than in 2003 (*p* < 0.05). Change is also apparent earlier in the day, with a higher proportion of respondents watching television in the 11:00 a.m. hour in 2019 relative to 2003 (*p* < 0.05). Thus, whereas late-night viewing decreased, viewing in the early evening and the late morning increased.

Paralleling the trend for weekdays, a smaller proportion of respondents watched television late at night on weekends/holidays in 2019 compared to 2003 (differences between 2003 and 2019 showed *p* < 0.01, from 10:00 p.m. through end of 1:00 a.m. hour). Similar to weekdays, there is a peak of difference in the 10:00 p.m. hour, when roughly 42 % of respondents watched TV in 2003, versus 38 % in 2019. However, this 4-percentage point difference is less than the 9-percentage point difference observed on weekdays.

In contrast to weekdays, increases on weekends/holidays (relative to 2003) in the probability of television viewership at particular hours are observed throughout a wider span of the entire daytime period. Similar to weekdays, the proportion of respondents watching television in the 7:00 p.m. hour also increased on weekends/holidays (*p* < 0.05 in Wald test). However, weekends/holidays also witnessed significant (*p* < 0.05) increases in the proportion of respondents watching television across the entire 6:00 a.m. through 12:00 p.m.-hour period.

When disaggregated by age (Fig. A2), statistically significant increases in daytime (and on weekdays, early evening) television viewing were only observed among 50–64-year-olds. However, a consistent finding across all age groups was a decrease in the proportion of respondents who watched television in the late evening on weekdays.

## 4. Discussion

This study provides evidence that during the period when video streaming rose in popularity and availability, younger adults shifted their television viewing to times that conflicted less with evening sleep schedules. The magnitude of these shifts was particularly large among adults ages 18–29, who by 2019 had stopped watching television roughly a half hour earlier on weekdays and 27 min earlier on weekends/holidays, compared to 2003 (when adjusting for covariates). Shifts to earlier television viewing end times were also observed among adults 30–49 and 50–64 years old. Paralleling trends found in an analysis of an ATUS subsample with different inclusion criteria (and through 2016 only) (Basner and Dinges, 2018), we also found that for adults under age 65, bedtimes shifted substantially earlier as time passed. Across both weekdays and weekends/holidays, bedtimes shifted at least 20 min earlier for adults ages 18–29, at least 15 min earlier for adults ages 30–49, and at least 7 min earlier for adults ages 50–64. The significance of a 20-min shift in bedtimes is underscored by the fact that among adults younger than age 65, the difference between weekday and weekend/holiday bedtimes over the observation period was 22 min (Supplementary Table A9).

In contrast, among adults 65 years of age and older, we did not observe a shift to earlier bedtimes or earlier cessation of evening television viewing from 2003 to 2019. This finding is perhaps related to the fact that near the end of our observation period, adults 65 and older were the least likely age group to use streaming as their primary means of watching television (Pew Research Center, 2017). In addition, changes in weekend/holiday bedtimes and television viewing end times were more modest among respondents 50–64 years old, another group that has been less likely to consider streaming as their main method of viewing television (Pew Research Center, 2017). Our results are potentially consistent with a scenario in which shifts in bedtimes and evening television viewing were diminished in older adults due to their lower uptake of streaming services to view television programming. This may in part be due to cohort trends in technology use and television viewing routines.

In adults under age 65, mediation analysis showed that the shift to earlier cessation of television viewing explained a substantial portion of the shift to earlier bedtimes—almost half of this shift, on both weekdays and weekends/holidays. These findings support the possibility that in the era of online video streaming, people are more able to schedule their television viewership at times facilitating healthy sleep schedules. Thus, it is plausible that online video streaming services could help people achieve earlier bedtimes.

Providing further support for the idea that streaming services allow people to schedule television viewing at times when they have less conflict with other activities, our findings suggest that in later years, adults under 65 years of age shifted the distribution of their television viewing across the week, such that an increasing proportion of viewing happened on weekends/holidays relative to weekdays. People under age 65 also shifted the distribution of their viewing across the 24-h day. Relative to 2003, by 2019, a smaller proportion of people under age 65 watched television late on weekday nights and, among 50–64-year-olds, a greater proportion watched television during the daytime (and, on weekdays, in the early evening). The significant decrease we observed in the proportion of respondents watching television late at night is consistent with prior research finding that the proportion of respondents watching television prior to bedtime fell from 2003 to 2016 (Basner and Dinges, 2018). These results suggest that people under age 65 increasingly appeared to be able to schedule television viewing at times that did not conflict with nighttime sleep.

It is important to note that access to streaming media varied across our observation period, but, in the ATUS data we analyze, we are unable to distinguish between streaming and non-streaming television viewing. As such, we were unable to directly determine if observed schedule changes were linked to video streaming. While we control for a host of sociodemographic characteristics in multivariable analyses, our results establish an associational—not causal—link between the expansion of video streaming services (assuming they expanded over time) and the timing of television viewership or bedtimes. Further, this study demonstrates only an associational link between the timing of television viewership and sleep schedules. Timing of television viewership and bedtimes might have changed for other reasons over this period, such as public awareness regarding the health importance of sleep (Basner and Dinges, 2018) or other potential omitted confounding factors net of streaming availability. Future research might exploit quasi-experimental methods to enhance causal inference regarding the relationship between more precise measures of both video streaming and sleep timing.

Major strengths of this study include national representation, 17 years of data, and the inclusion of 24-h time diaries. However, a limitation of analyzing time diaries collected over many years is that measurement differences potentially could have affected how television viewership was captured over the observed period. Guidelines for ATUS coders of time diary activities has changed over time, likely reflecting societal changes in video viewing habits. For example, in the ATUS coding system, “watching videos on YouTube” first appeared as an example of an activity that should be coded as “Television and movies (not religious)” in 2011 (U.S. Bureau of Labor Statistics, 2024). However, throughout the full period, coding rules consistently specified that watching video in a home should be classified under “Television and movies (not religious).” A separate but important note regarding measurement of television viewing is that the ATUS focus on primary activities restricts our ability to capture television viewing as a secondary activity that people carry out simultaneously while engaged in other tasks. Another limitation of this analysis tracking change over time is that as people shifted from viewing video on traditional television sets to devices such as tablets and smartphones, respondents may have changed how they report on watching television in bed. It is possible that respondents could have spent time watching videos on their phone in bed and erroneously reported this as sleeping, potentially leading to the appearance of earlier bedtimes as people streamed more video online.

We note that schedule constraints are not always clearly differentiated between weekdays and weekends/holidays (for example, some people work on weekends). Thus, future research might examine additional nuance regarding how contemporary television viewing has shifted around individual schedule constraints. In addition, with the ATUS data, we are unable to examine the same respondent across both a weekday and a weekend/holiday, which limits our ability to make inferences at the individual level about how the distribution of television viewing shifted between weekdays and weekends/holidays. Future research using multiple days of time diary data collection would be useful to address this issue.

An additional limitation of our study is that given our bedtime measurement technique, our sample likely does not include certain respondents with irregular or shifted sleep schedules, such as shift workers. Future research might examine sleep throughout a wider span of the day or objective sleep measures. Time diary report of daily activities is not an objective form of measurement. However, research shows that time diary reports of television watching are correlated with television watching as captured via camera (Gershuny et al., 2020), and time diary reports of sleep are correlated with sleep as captured via camera (Gershuny et al., 2020) and as measured objectively using a wearable device (Kaplan et al., 2019).

We raise the important point that this research focuses on bedtimes, one aspect of sleep health. The introduction of streaming may have had negative effects on other dimensions of sleep, such as poorer sleep quality, increased insomnia symptoms, or increased fatigue due to more binge-watching (Exelmans & Van den Bulck, 2017)—or poorer sleep quality if streaming contributed to increased cell phone use at bedtime (Exelmans & Van den Bulck, 2016). It will be important for future work to investigate such potential tradeoffs in how the growth of streaming has affected sleep. It will also be important for future work to address whether the effects of streaming across distinct age groups differed in more recent years. Finally, our results are specific to the United States where high-speed internet access, which could support use of streaming services, is distributed unequally (Anderson and Perrin, 2018; Vogels, 2021). Future research might expand this work to determine how streaming has affected sleep in other countries across the digital divide.

## 5. Conclusion

Sleep is an important component of health promoting lifestyles (Krueger et al., 2011; Saint Onge and Krueger, 2017), with late bedtimes linked to increased BMI (Asarnow et al., 2015) and mortality risk (Wang et al., 2021). Public health efforts aim to improve sleep (Office of Disease Prevention and Health Promotion), but sleep improvements are frequently compromised by competing and immutable demands such as child-rearing and work hours (Krueger et al., 2023; Meyer, 2024). This research represents a step forward in understanding the extent that television viewing in the streaming era may represent a potential area of sleep intervention. We found that for adults under age 65, television viewing schedules significantly changed from 2003 to 2019, a period of widespread streaming expansion. For adults under age 65, as time passed, the proportion of television viewed on weekends/holidays (relative to weekdays) increased, and, by the end of this period, a lower percentage of people watched television at late evening hours. We offer evidence that online video streaming may have potential to promote healthy sleep timing through a form of time shifting (Wajcman, 2008), by showing that the recent shifts to earlier cessation of evening television viewing account for a substantial portion of recent shifts to earlier bedtimes. Public health messaging might leverage the relatively open scheduling possibilities provided with streaming to encourage moderate television watching at times that avoid conflict with healthier sleep schedules. While individuals may be unwilling to reduce time spent in preferred leisure activities, there is potential for technology change to offer more control over enjoying these activities while also achieving earlier bedtimes.

## Supplementary Material

MMC1

Appendix A. Supplementary data

Supplementary data to this article can be found online at https://doi.org/10.1016/j.socscimed.2025.117977.

## Data Availability

ATUS data are available from the IPUMS ATUS Extract Builder (https://www.atusdata.org/atus/). Interested parties may consult that website for additional information on access.
