# Supplementary material for: The role of shifted television viewing on earlier bedtimes in the streaming era
Source: Soc Sci Med. Author manuscript; Available in PMC 2025 Oct 15. (PMC12527564; doi:10.1016/j.socscimed.2025.117977)
Supplement: MMC1 [file NIHMS2102058-supplement-MMC1.docx]

**APPENDIX A.** Online Supplement

**Table A1.** Full Sample Size by Year, Adult ATUS Respondents from Years 2003 to 2019

| Year | N |
| --- | --- |
| 2003 | 19,216 |
| 2004 | 12,950 |
| 2005 | 12,098 |
| 2006 | 11,871 |
| 2007 | 11,306 |
| 2008 | 11,788 |
| 2009 | 12,275 |
| 2010 | 12,312 |
| 2011 | 11,647 |
| 2012 | 11,675 |
| 2013 | 10,692 |
| 2014 | 10,891 |
| 2015 | 10,279 |
| 2016 | 9,867 |
| 2017 | 9,653 |
| 2018 | 9,083 |
| 2019 | 8,966 |
| Total | 196,569 |

**Table A2.** Descriptive Statistics for Full Sample in Years 2003 and 2019, Adult ATUS Respondents

|  | 2003 | 2019 |
| --- | --- | --- |
| Mean Bedtime (Clock Time) | 10:44 p.m. | 10:29 p.m. |
|  | (2 hours 10 minutes) | (1 hour 20 minutes) |
| Mean Amount of Television Watched (Hours) | 2.6 | 2.9 |
|  | (3.7) | (2.6) |
| Mean Age (Years) | 45.5 | 47.9 |
|  | (23.5) | (15.6) |
| Male (%) | 47.7 | 48.1 |
| Race/Ethnicity (%) |  |  |
| Non-Hispanic White | 72.6 | 65.6 |
| Hispanic | 12.3 | 16.4 |
| Non-Hispanic Black | 10.7 | 11.7 |
| Other | 4.5 | 6.3 |
| Education (%) |  |  |
| < High School | 15.4 | 9.6 |
| High School | 32.4 | 28.7 |
| Some College or Associate's | 26.3 | 25.4^a^ |
| College and above | 25.9 | 36.3 |
| Number of Children in Household (%) |  |  |
| Zero | 60.6 | 66.8 |
| One | 16.5 | 14.5 |
| Two | 14.6 | 11.9 |
| Three or More | 8.3 | 6.9 |
| Partnership Status |  |  |
| Living with Spouse | 58.8 | 53.7 |
| Living with Unmarried Partner | 4.6 | 5.9 |
| Not Living with Partner | 36.5 | 40.4 |
| Born Outside U.S. (%) | 13.7 | 18.0 |
| Mean Hours Worked on Diary Day | 3.5 | 3.4^a^ |
|  | (5.9) | (3.7) |
| N | 19,216 | 8,966 |

Source: American Time Use Survey, 2003 and 2019.

Note: Generated using sampling weights. Standard deviations for continuous variables are shown in parentheses.

^a^Difference with 2003 is *not* statistically significant at *p* < 0.05, as assessed with bivariate regression on a dichotomous indicator for year (linear regression for continuous variables and logistic regression for dichotomous indicators).

**Table A3.** Descriptive Statistics for “Evening Television” Subsample in Years 2003 and 2019, Adult ATUS Respondents who Viewed Television in Evening

|  | 2003 | 2019 |
| --- | --- | --- |
| Mean Bedtime (Clock Time) | 10:44 p.m. | 10:29 p.m. |
|  | (2 hours 1 minute) | (1 hour 16 minutes) |
| Mean Television Viewing End Time | 10:10 p.m. | 9:57 p.m. |
|  | (2 hours 26 minutes) | (1 hour 32 minutes) |
| Mean Amount of Television Watched (Hours) | 3.4 | 3.8 |
|  | (3.6) | (2.7) |
| Mean Age (Years) | 46.7 | 49.9 |
|  | (23.9) | (16.0) |
| Male (%) | 49.0 | 48.9^a^ |
| Race/Ethnicity (%) |  |  |
| Non-Hispanic White | 73.0 | 67.9 |
| Hispanic | 12.4 | 15.4 |
| Non-Hispanic Black | 10.3 | 11.3 |
| Other | 4.3 | 5.4 |
| Education (%) |  |  |
| < High School | 15.5 | 9.3 |
| High School | 33.8 | 29.9 |
| Some College or Associate's | 25.8 | 25.0^a^ |
| College and above | 24.9 | 35.8 |
| Number of Children in Household (%) |  |  |
| Zero | 62.9 | 69.4 |
| One | 15.9 | 13.6 |
| Two | 13.7 | 10.8 |
| Three or More | 7.6 | 6.2 |
| Partnership Status |  |  |
| Living with Spouse | 59.1 | 54.5 |
| Living with Unmarried Partner | 4.5 | 6.0 |
| Not Living with Partner | 36.3 | 39.4 |
| Born Outside U.S. (%) | 13.3 | 16.5 |
| Hours Worked on Diary Day | 3.2 | 3.1^a^ |
|  | (5.6) | (3.6) |
| N | 14,205 | 6,763 |

Source: American Time Use Survey, 2003 and 2019.

Note: Generated using sampling weights. Standard deviations for continuous variables are shown in parentheses.

^a^Difference with 2003 is *not* statistically significant at *p* < 0.05, as assessed with bivariate regression on a dichotomous indicator for year (linear regression for continuous variables and logistic regression for dichotomous indicators).

**Table A4.** Selected Coefficients, Fit Statistics, and Likelihood Ratio Test Statistics from Unweighted Ordinary Least Squares Regression Models Predicting **Bedtime** among Adult ATUS Respondents who Viewed Television in Evening, Years 2003 to 2019

| Model | Years Since 2003 (Coefficient) | Years Since 2003^2^ (Coefficient) | Years Since 2003^3^ (Coefficient) | AIC | BIC | Log Likelihood | Likelihood Ratio Test Statistic | df | p-value |
| --- | --- | --- | --- | --- | --- | --- | --- | --- | --- |
| Weekdays |  |  |  |  |  |  |  |  |  |
| Linear | -0.78*** |  |  | 1128580.0 | 1128845.0 | -564262 | 3452.41 | 27 | 0.0000 |
| Linear, Quadratic | -0.09 | -0.05*** |  | 1128568.0 | 1128843.0 | -564255.2 | 13.64 | 1 | 0.0002 |
| Linear, Quadratic, Cubed | 0.31 | -0.11 | 0.00 | 1128569.0 | 1128854.0 | -564254.7 | 1.02 | 1 | 0.3120 |
| Weekends/Holidays |  |  |  |  |  |  |  |  |  |
| Linear | -1.08*** |  |  | 609972.4 | 610202.6 | -304960.2 | 2399.84 | 25 | 0.0000 |
| Linear, Quadratic | -0.95*** | -0.01 |  | 609974.2 | 610213.2 | -304960.1 | 0.24 | 1 | 0.6268 |
| Linear, Quadratic, Cubed | -0.32 | -0.12 | 0.00 | 609975.0 | 610222.9 | -304959.5 | 1.19 | 1 | 0.2753 |

Source: American Time Use Survey, 2003-2019.

Note: Coefficients are shown in minutes past midnight on the diary day. Sampling weights not used. All models control for region, season, age (and age squared), sex, race/ethnicity, education, number of children in the household, partnership status, nativity, and hours worked on the diary day. The weekday model controls for day of the week (Sunday, Monday, Tuesday, Wednesday, or Thursday), and the weekend/holiday model controls for whether the diary day was a non-holiday Friday, non-holiday Saturday, or holiday.

**p* < .05, ***p* < .01, ****p* < .001 (two-tailed tests).

**Table A5.** Selected Coefficients, Fit Statistics, and Likelihood Ratio Test Statistics from Unweighted Ordinary Least Squares Regression Models Predicting **End Time of Television Viewing** among Adult ATUS Respondents who Viewed Television in Evening, Years 2003 to 2019

| Model | Years Since 2003 (Coefficient) | Years Since 2003^2^ (Coefficient) | Years Since 2003^3^ (Coefficient) | AIC | BIC | Log Likelihood | Likelihood Ratio Test Statistic | df | p-value |
| --- | --- | --- | --- | --- | --- | --- | --- | --- | --- |
| Weekdays |  |  |  |  |  |  |  |  |  |
| Linear | -0.82*** |  |  | 1167870.0 | 1168136.0 | -583907.1 | 2126.79 | 27 | 0.0000 |
| Linear, Quadratic | -0.14 | -0.05** |  | 1167863.0 | 1168138.0 | -583902.6 | 9.11 | 1 | 0.0025 |
| Linear, Quadratic, Cubed | -0.08 | -0.05 | 0.00 | 1167865.0 | 1168149.0 | -583902.6 | 0.01 | 1 | 0.9112 |
| Weekends/Holidays |  |  |  |  |  |  |  |  |  |
| Linear | -0.89*** |  |  | 631134.8 | 631365.0 | -315541.4 | 1143.96 | 25 | 0.0000 |
| Linear, Quadratic | -1.14*** | 0.02 |  | 631136.2 | 631375.3 | -315541.1 | 0.60 | 1 | 0.4369 |
| Linear, Quadratic, Cubed | -0.23 | -0.14 | 0.01 | 631136.6 | 631384.5 | -315540.3 | 1.64 | 1 | 0.2001 |

Source: American Time Use Survey, 2003-2019.

Note: Coefficients are shown in minutes past midnight on the diary day. Sampling weights not used. All models control for region, season, age (and age squared), sex, race/ethnicity, education, number of children in the household, partnership status, nativity, and hours worked on the diary day. The weekday model controls for day of the week (Sunday, Monday, Tuesday, Wednesday, or Thursday), and the weekend/holiday model controls for whether the diary day was a non-holiday Friday, non-holiday Saturday, or holiday.

**p* < .05, ***p* < .01, ****p* < .001 (two-tailed tests).

**Table A6.** Selected Coefficients from Ordinary Least Squares Regression Models Predicting Bedtime and Television Viewing End Time (in Minutes) among ATUS Respondents 18 to 64 Years Old who Viewed Television in Evening, Disaggregated by Age (Years 2003 to 2019)

|  | Weekdays | |  | Weekends/Holidays | |
| --- | --- | --- | --- | --- | --- |
|  | Bedtime | TV End Time |  | Bedtime | TV End Time |
| **Panel A. Ages 18-29** |  |  |  |  |  |
| Years Since 2003 | -0.03 | 0.40 |  | -1.99*** | -1.70*** |
|  | (0.83) | (1.01) |  | (0.36) | (0.46) |
| Years Since 2003^2^ | -0.11* | -0.14* |  |  |  |
|  | (0.05) | (0.06) |  |  |  |
| Constant | 1362.15*** | 1306.84*** |  | 1399.17*** | 1330.57*** |
|  | (6.34) | (8.55) |  | (9.17) | (12.40) |
| N | 11,789 | 11,789 |  | 6,180 | 6,180 |
| R-squared | .06 | .03 |  | .09 | .02 |
| **Panel B. Ages 30-49** |  | |  |  | |
| Years Since 2003 | -0.03 | -0.29 |  | -1.29*** | -1.49*** |
|  | (0.37) | (0.49) |  | (0.17) | (0.21) |
| Years Since 2003^2^ | -0.07** | -0.08* |  |  |  |
|  | (0.02) | (0.03) |  |  |  |
| Constant | 1334.86*** | 1286.97*** |  | 1402.29*** | 1348.55*** |
|  | (9.42) | (10.67) |  | (14.67) | (17.60) |
| N | 36,782 | 36,782 |  | 20,323 | 20,323 |
| R-squared | .05 | .03 |  | .05 | .03 |
| **Panel C. Ages 50-64** |  | |  |  | |
| Years Since 2003 | 0.68 | 0.44 |  | -0.96*** | -0.60** |
|  | (0.50) | (0.53) |  | (0.21) | (0.23) |
| Years Since 2003^2^ | -0.12*** | -0.09** |  |  |  |
|  | (0.03) | (0.03) |  |  |  |
| Constant | 1444.80*** | 1337.26*** |  | 1274.33*** | 1164.52*** |
|  | (63.05) | (73.63) |  | (89.16) | (109.42) |
| N | 25,179 | 25,179 |  | 13,398 | 13,398 |
| R-squared | .04 | 0.02 |  | .04 | .02 |
| **Panel D. Ages 65 and older** | | |  |  | |
| Years Since 2003 | -0.53 | -0.31 |  | -0.22 | 0.28 |
|  | (0.48) | (0.57) |  | (0.18) | (0.23) |
| Years Since 2003^2^ | 0.01 | 0.02 |  |  |  |
|  | (0.03) | (0.03) |  |  |  |
| Constant | 1401.33*** | 1226.81*** |  | 1218.01*** | 1404.04*** |
|  | (60.21) | (78.54) |  | (90.42) | (97.37) |
| N | 22,936 | 22,936 |  | 11,791 | 11,791 |
| R-squared | .03 | .02 |  | .03 | .03 |

Source: American Time Use Survey, 2003-2019.

Note: TV = Television Viewing. Coefficients are shown in minutes past midnight on the diary day. Generated using sampling weights. All models control for region, season, age (and age squared), sex, race/ethnicity, education, number of children in the household, partnership status, nativity, and hours worked on the diary day. The weekday model controls for day of the week (Sunday, Monday, Tuesday, Wednesday, or Thursday), and the weekend/holiday model controls for whether the diary day was a non-holiday Friday, non-holiday Saturday, or holiday.

†*p* < .10, **p* < .05, ***p* < .01, ****p* < .001 (two-tailed tests).

**Table A7.** Selected Coefficients from Ordinary Least Squares Regression Models Predicting Bedtime (in Minutes) among ATUS Respondents 18 to 64 Years Old who Viewed Television in Evening, Years 2003 to 2019

|  | Weekdays | |  | Weekends/Holidays | |
| --- | --- | --- | --- | --- | --- |
|  | Model 1  (No control for TV End Time) | Model 2  (Control for TV End Time) |  | Model 1  (No control for TV End Time) | Model 2  (Control for TV End Time) |
| Years Since 2003 | 0.06 | 0.22 |  | -1.41*** | -0.72*** |
|  | (0.29) | (0.23) |  | (0.13) | (0.11) |
| Years Since 2003^2^ | -0.09*** | -0.05*** |  |  |  |
|  | (0.02) | (0.01) |  |  |  |
| Television Viewed (Hours) | 4.69*** | -1.05*** |  | 4.03*** | -0.74*** |
|  | (0.20) | (0.15) |  | (0.27) | (0.20) |
| Television Viewing End Time (Minutes) |  | 0.53*** |  |  | 0.47*** |
|  |  | (0.01) |  |  | (0.01) |
| Constant | 1,344.31*** | 677.89*** |  | 1,375.48*** | 768.65*** |
|  | (2.87) | (8.70) |  | (4.28) | (13.01) |
| N | 73,750 | 73,750 |  | 39,901 | 39,901 |
| R-squared | .07 | .43 |  | .08 | .37 |

Source: American Time Use Survey, 2003-2019.

Note: TV = Television Viewing. Coefficients are shown in minutes past midnight on the diary day. Generated using sampling weights. All models control for region, season, age (and age squared), sex, race/ethnicity, education, number of children in the household, partnership status, nativity, and hours worked on the diary day. The weekday model controls for day of the week (Sunday, Monday, Tuesday, Wednesday, or Thursday), and the weekend/holiday model controls for whether the diary day was a non-holiday Friday, non-holiday Saturday, or holiday.

†*p* < .10, **p* < .05, ***p* < .01, ****p* < .001 (two-tailed tests).

**Table A8.** Results from Mediation Analysis of Evening Television Viewing End Time (in Minutes) as Mediator of Change in Bedtime (in Minutes) over Time among ATUS Respondents 18 to 64 Years Old who Viewed Television in Evening, Years 2003 to 2019

|  | Estimate | se | z | p-value |
| --- | --- | --- | --- | --- |
| Weekdays (Independent Variable = Years Since 2003^2^) |  |  |  |  |
| Indirect effect via Television Viewing End Time | -0.04 | 0.01 | -3.40 | 0.001 |
| Direct effect | -0.05 | 0.02 | -3.35 | 0.001 |
| Total effect | -0.09 | 0.02 | -4.99 | 0.000 |
| Sobel test | -0.04 | 0.01 | -3.40 | 0.001 |
| Proportion of Total Effect that Is Mediated | 0.448 |  |  |  |
| Weekends/Holidays (Independent Variable = Years Since 2003) |  |  |  |  |
| Indirect effect via Television Viewing End Time | -0.69 | 0.08 | -9.15 | 0.000 |
| Direct effect | -0.72 | 0.11 | -6.80 | 0.000 |
| Total effect | -1.41 | 0.13 | -10.56 | 0.000 |
| Sobel test | -0.69 | 0.08 | -9.15 | 0.000 |
| Proportion of Total Effect that Is Mediated | 0.488 |  |  |  |

Source: American Time Use Survey, 2003-2019.

Note: Generated using sampling weights. N = 73,750 for weekdays and 39,901 for weekends/holidays. All models control for region, season, age (and age squared), sex, race/ethnicity, education, number of children in the household, partnership status, nativity, hours worked on the diary day, and amount of television viewed on the diary day. The weekday model controls for day of the week (Sunday, Monday, Tuesday, Wednesday, or Thursday), and the weekend/holiday model controls for whether the diary day was a non-holiday Friday, non-holiday Saturday, or holiday. The weekday model also includes a linear term for “years since 2003.”

**Table A9.** Bedtime for Sample of ATUS Respondents 18 to 64 Years Old on Weekdays and Weekends/Holidays, Years 2003 to 2019

|  | Weekdays | Weekends/Holidays |
| --- | --- | --- |
| Bedtime (Clock Time) | 10:35 p.m. | 10:57 p.m. |
|  | (1 hour 28 minutes) | (1 hour 49 minutes) |
| N | 99,623 | 55,707 |

Source: American Time Use Survey, 2003-2019.

Note: Generated using sampling weights. Standard deviations are shown in parentheses. Friday and Saturday are considered “weekend.”

**Figure A1**. Ratio of Weekend/Holiday to Weekday Television by Year and Age Category, Years 2003 to 2019 (ATUS Respondents 18 to 64 Years Old with Valid Bedtime)


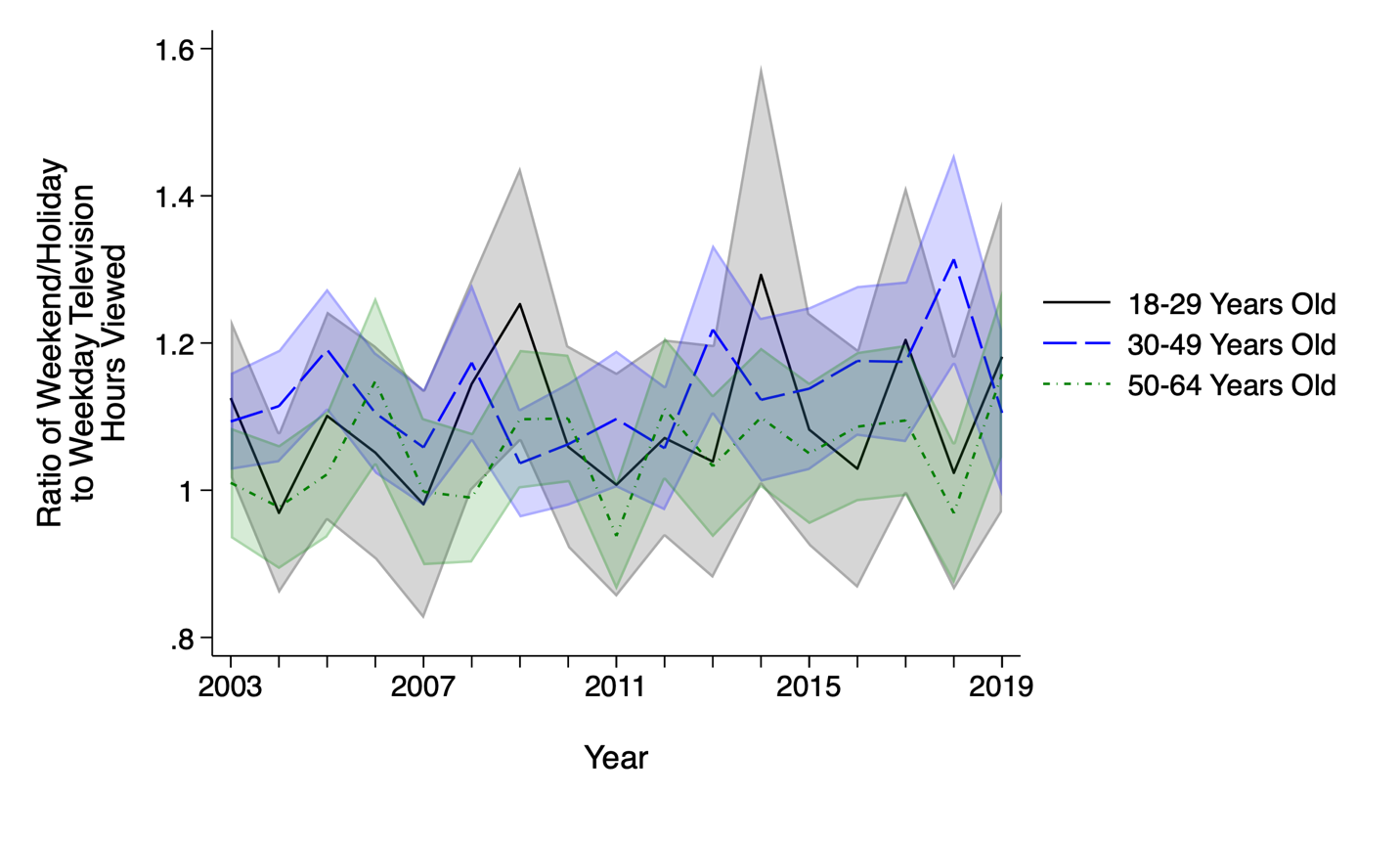


Source: American Time Use Survey, 2003-2019.

Note: Lines show descriptive statistics generated using sampling weights at the individual level. Shaded areas show 95% confidence intervals. N = 155,330 (26,253 for ages 18-29, 79,496 for ages 30-49, 49,581 for ages 50-64).


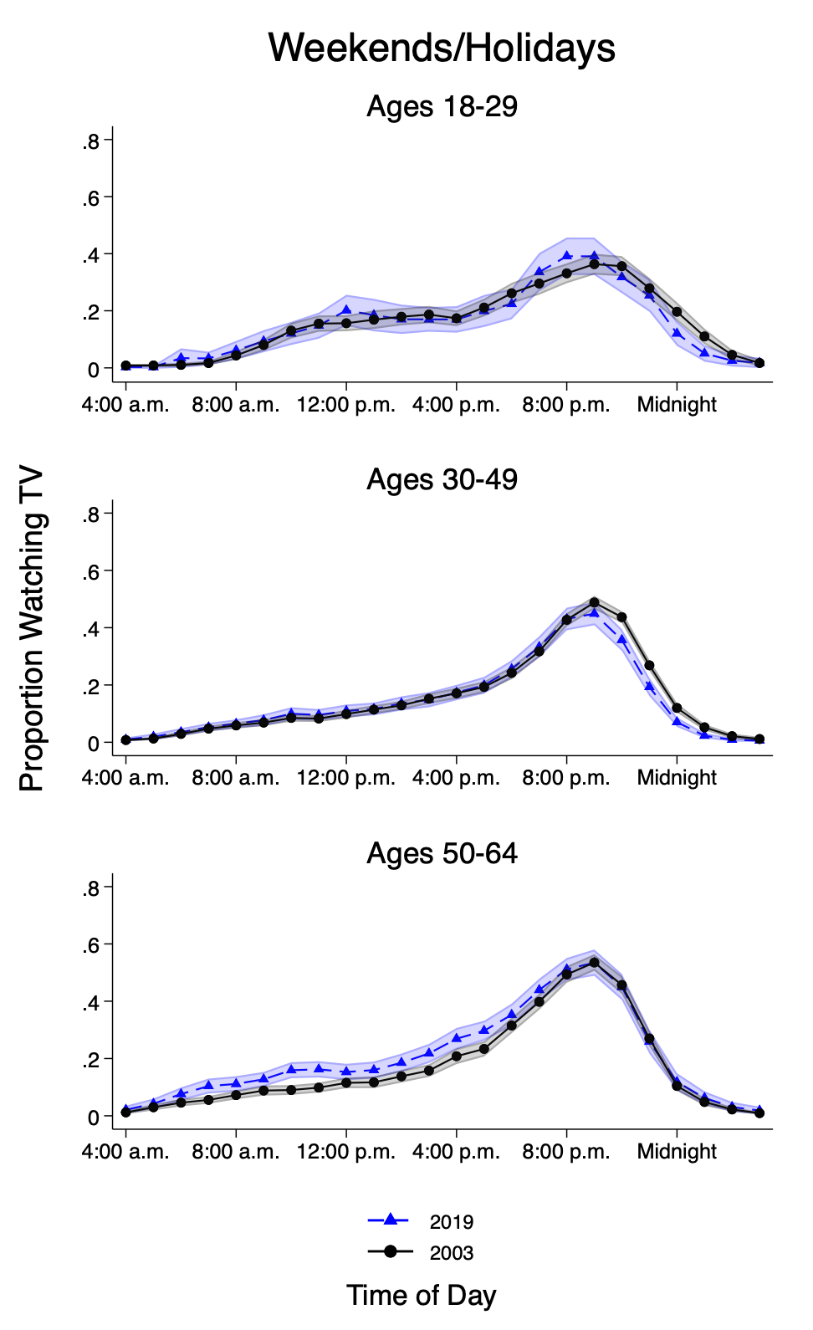

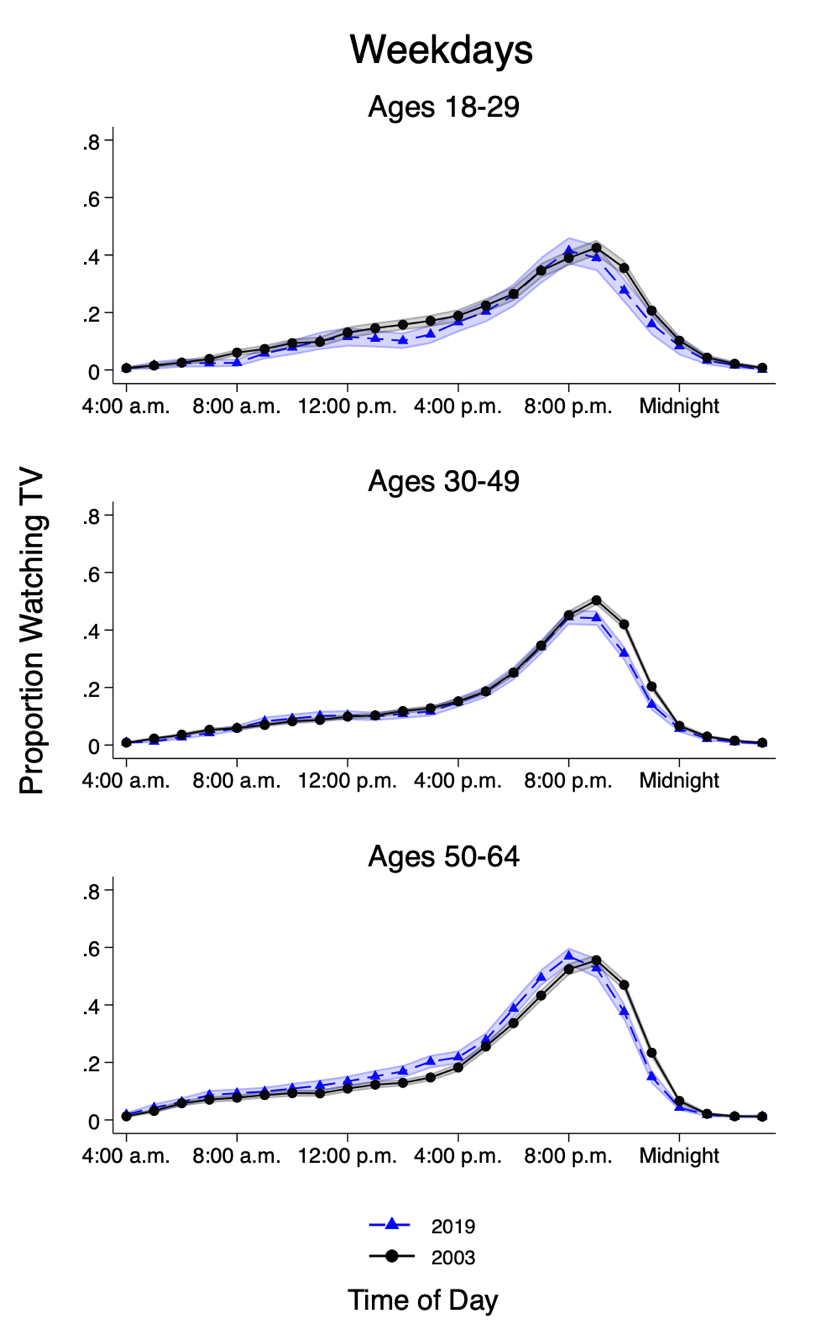
**Figure A2**. Proportion that Viewed Television within Each Hour of the Day by Age, Year 2003 versus 2019 (ATUS Respondents 18 to 64 Years Old with Valid Bedtime)

Source: American Time Use Survey, 2003 and 2019.

Note: TV = television. Shaded areas show 95% confidence intervals. Generated using sampling weights. N = 22,212 individuals (N = 10,115 for weekdays in 2003 [1,734 for ages 18-29, 5,532 for ages 30-49, and 2,849 for ages 50-64], 5,669 for weekends/holidays in 2003 [1,033 for ages 18-29, 3,010 for ages 30-49, and 1,626 for ages 50-64], 4,158 for weekdays in 2019 [638 for ages 18-29, 1,992 for ages 30-49, and 1,528 for ages 50-64], and 2,270 for weekends/holidays in 2019 [361 for ages 18-29, 1,079 for ages 30-49, and 830 for ages 50-64]).
